# Transformations of the macromolecular landscape at mitochondria during DNA-damage-induced apoptotic cell death

**DOI:** 10.1038/cddis.2014.405

**Published:** 2014-10-09

**Authors:** N Yadav, A Pliss, A Kuzmin, P Rapali, L Sun, P Prasad, D Chandra

**Affiliations:** 1 Department of Pharmacology and Therapeutics, Center for Genetics and Pharmacology, Roswell Park Cancer Institute, Elm and Carlton Streets, Buffalo, NY, USA; 2 Institute for Lasers, Photonics and Biophotonics, University at Buffalo, State University of New York, Buffalo, NY, USA; 3 Gastrointestinal Division, Sir Run Run Shaw Hospital, Zhejiang University Medical School, Hangzhou, China

## Abstract

Apoptosis is a dynamic process regulated by mitochondrion critical for cellular respiration and survival. Execution of apoptosis is mediated by multiple protein signaling events at mitochondria. Initiation and progression of apoptosis require numerous apoptogenic factors that are either released from or sequestered in mitochondria, which may transform the biomolecular makeup of the organelle. In this communication, using Raman microspectroscopy, we demonstrate that transformation in biomolecular composition of mitochondrion may be used as apoptosis marker in an individual cell. For the first time, we show that significant changes occur in the concentrations of RNA, DNA, protein, and lipid constituents of mitochondria during apoptosis. The structural analysis of proteins on mitochondria demonstrated a decrease in *α*-helix secondary structure content, and an increase in the levels of random coils and *β*-sheets on mitochondria. This may represent an additional hallmark of apoptosis. Strikingly, we observed nearly identical changes in macromolecular content of mitochondria both in the presence and absence of a key proapoptotic protein, Bax (Bcl-2-associated X protein). Increased DNA level in mitochondria corresponded with higher mitochondrial DNA (mtDNA), cellular reactive oxygen species (ROS), and mitochondrial ROS production. Upregulation of polymerase-*γ* (POLG), mitochondrial helicase Twinkle, and mitochondrial transcription factor A (Tfam) in response to DNA damage correlated with increased mtDNA and RNA synthesis. Elevated activity of oxidative phosphorylation complexes supports functional mitochondrial respiration during apoptosis. Thus, we define previously unknown dynamic correlation of macromolecular structure of mitochondria and apoptosis progression in the presence and absence of Bax protein. These findings open up a new approach for monitoring physiological status of cells by non invasive single-cell method.

Apoptosis, a form of programmed cell death, is a stepwise process essential for normal tissue function and homeostasis. Dysregulated apoptosis intimately associates with the development of cancer, immune disorders, neurodegeneration, and cardiac diseases.^1^ Although significant progress has been made in understanding the process of apoptosis *per se*, the identification of apoptotic or diseased cells under physiological conditions is not defined. Therefore, there is need for new approaches capable of identifying and monitoring the dynamically changing cellular structure during the progression of apoptosis.

The initiation and execution of apoptosis is mediated through major cellular organelle, mitochondrion. Mitochondria produce ATP via cellular respiration, participate in regulation of cellular homeostasis, as well as initiation of apoptosis. The process of apoptosis is dependent on completion of multiple complex signaling events orchestrated around mitochondria, which ultimately activate caspases to execute apoptotic cell death.^2,3^ Thus, execution of apoptosis involves the transformation of biomolecular makeup of mitochondria. Bcl-2 (B-cell lymphoma 2) family proteins, which are broadly classified as prosurvival, and proapoptotic proteins regulate the mitochondrial integrity. Upon receiving apoptotic signal, Bax, a proapoptotic member of Bcl-2 protein family, which normally localizes to the cytosolic compartment, translocates to and oligomerizes on the outer mitochondrial membrane (OMM) to form the channels through the membrane. Bax can homo-oligomerize to form Bax channel or hetero-oligomerize with another proapoptotic protein Bak (Bcl-2 antagonist/killer) to form Bax/Bak channel.^4,5^ The channel formation promotes permeability of mitochondria leading to the release of mitochondrial cytochrome *c*, which triggers apoptosis.^6,7^

It is important to emphasize that these signaling pathways have been studied in mitochondria isolated from a population of cells, which may not represent cascade of events happening in live individual cells. In addition, very little is understood about the sequential timing of structural reorganization of mitochondrion throughout the apoptosis process. It has been increasingly recognized that monitoring of mitochondria structure and function is essential for mitochondria quality control assessment.^8^ Thus, characterization of mitochondria at the molecular level will provide valuable insight into the physiological state and function of both normal and diseased cells. To evaluate physiological status of cells under normal and stress conditions, we introduce the analysis of confocal Raman spectroscopic signals for probing the biochemical content of mitochondria. Different molecular bonds produce their characteristic spectra, enabling selective identification of protein, RNA, DNA, and lipid in the biological samples. The intensity of Raman scattering response is linearly dependent on the concentration of a particular type of molecule in the probe that allows for quantitative concentration measurements of these biomolecules *in situ*. Moreover, the Raman spectra identify changes in the secondary structural composition of proteins.^9, 10, 11^

We analyzed the global structural organization of mitochondria and identified concentrations of major classes of macromolecules such as protein, DNA, RNA, and lipid in mitochondria during DNA-damage-induced apoptosis. We observed DNA and protein accumulation on mitochondria, whereas the levels of RNA and lipid were reduced. The pattern of protein conformational structure was altered during the process of apoptosis in a similar manner in the absence and presence of Bax. We observed substantial increase of mitochondrial DNA (mtDNA) concentration, cellular reactive oxygen species (ROS), and mitochondrial ROS production in response to DNA damage in both types of cells. These findings clearly implicate that mitochondrial function is critical for DNA-damage-induced apoptotic cell death both in the presence and absence of Bax.

## Results

### Doxorubicin induces apoptotic cell death in colon cancer cells

**Figure 1:**
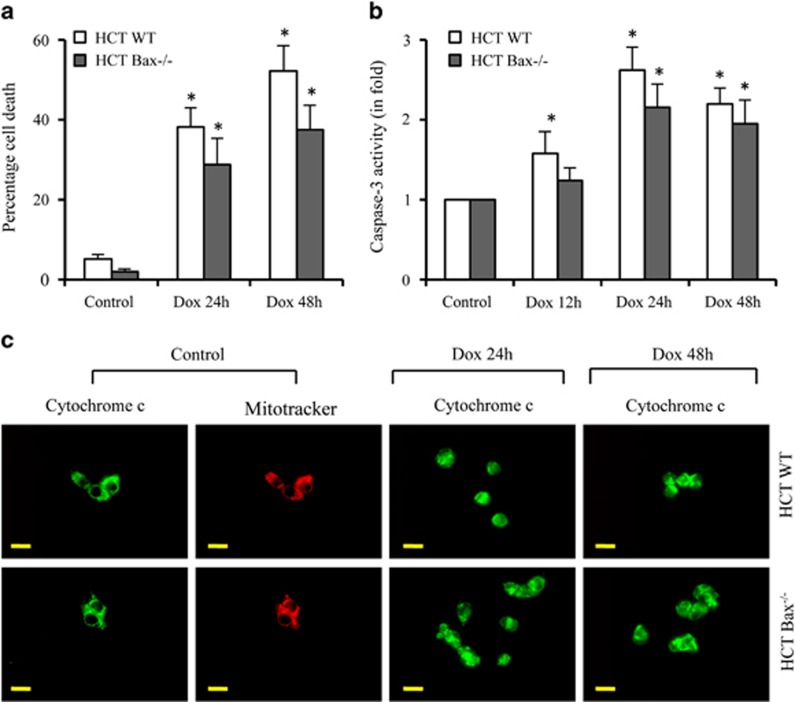
Dox induces caspase-dependent apoptosis irrespective of Bax status. (**a** and **b**) HCT116 WT and HCT116 *Bax*^*−/−*^ cells were treated with Dox (10 *μ*M) for indicated times. Percentage cell death was quantified using Trypan blue method (**a**). Equal amounts of protein were used for DEVDase activity (caspase-3) measurement (**b**). (**c**) HCT116 WT and HCT116 *Bax*^*−/−*^ cells were treated with Dox (10 *μ*M) for indicated times, cells were labeled live with MitoTracker Orange to label mitochondria and immunolabeled for cytochrome *c*. Representative micrographs are shown and magnification bars represent 20 *μ*m. Dox, doxorubicin; HCT, HCT116. Data are mean±S.D., *n*=3; *P<0.05

To understand the dynamic reorganization of macromolecular structure of mitochondria during apoptosis, we used doxorubicin (Dox), a commonly known anticancer and DNA-damaging agent, for triggering apoptosis in HCT116 colon cancer cells. We observed ~40–50% cell death between 24 and 48 h in response to Dox using Trypan blue staining (Figure 1a). We also observed that 40–50% of cells showed Annexin V labeling (data not shown), suggesting that Dox induces apoptotic cell death. Dox-induced caspase activity further suggests the involvement of caspase-dependent apoptosis in HCT116 colon cancer cells (Figure 1b).

### In the absence of Bax, Bak supports Dox-induced apoptosis

During apoptosis, proapoptotic multidomain Bcl-2 family proteins, Bax and Bak, oligomerize on mitochondria to form channels on mitochondrial membrane causing the leakage of apoptogenic proteins such as cytochrome *c*.^12, 13, 14, 15^ To investigate the impact of Bax deficiency in DNA-damage-induced apoptosis, we evaluated Dox-induced apoptosis in isogenic Bax-deficient HCT116 colon cancer cells. We observed that Dox induces cell death and apoptosis in both HCT116 *Bax*^*−/−*^ and HCT116 WT cells (Figure 1a), although lower levels of cell death were observed in Bax-deficient cells. Similarly, caspase activation was also observed in both types of cells (Figure 1b), suggesting that caspase-dependent apoptosis contributes to apoptotic cell death both in the presence and absence of Bax.

### Dox induces cytochrome *c* release in the presence and absence of Bax

As Bax deletion did not inhibit DNA-damage-induced caspase activation and apoptotic cell death, we investigated whether the absence of Bax modulates the mitochondrial membrane permeabilization. We observed diffused cytochrome *c* labeling in both types of cells (Figure 1c), suggesting that the lack of Bax did not inhibit cytochrome *c* release during Dox-induced apoptosis. Since the spectrum of Dox overlaps with MitoTracker Orange or Red, we could not capture MitoTracker labeling to mitochondria in Dox-treated cells. These findings suggest that DNA-damaging agent Dox induces permeabilization of the mitochondrial membrane in the presence and absence of Bax.

### Similar levels of proteins, DNA, and RNA but not lipids accumulate on mitochondria in HCT116 WT and Bax-deficient cells

Extensive amount of research has been performed focusing on cellular protein signaling; however, the impact of combined effects of protein, lipid, DNA, and RNA on apoptosis have not been clearly defined. To determine how overall biomolecular makeup of mitochondria is changed in response to DNA-damaging agent, we adopted Raman spectroscopy to quantify the levels of protein, DNA, RNA, and lipid. Mitochondria were labeled with MitoTracker Green FM and the sites for Raman spectra acquisition were targeted by the fluorescence signal from the MitoTracker. Based on averaged dimensions of mitochondria as an ellipse of 0.5–1 *μ*m in diameter and ~2 *μ*m in height, the focused Raman laser probes at volume equivalent to 1–2 mitochondria for each measurement. We first obtained Raman spectra of mitochondria in WT and Bax-deficient HCT116 cells both untreated and upon Dox treatment by selecting seven or more representative cells (Supplementary Figure S1).

**Figure 2:**
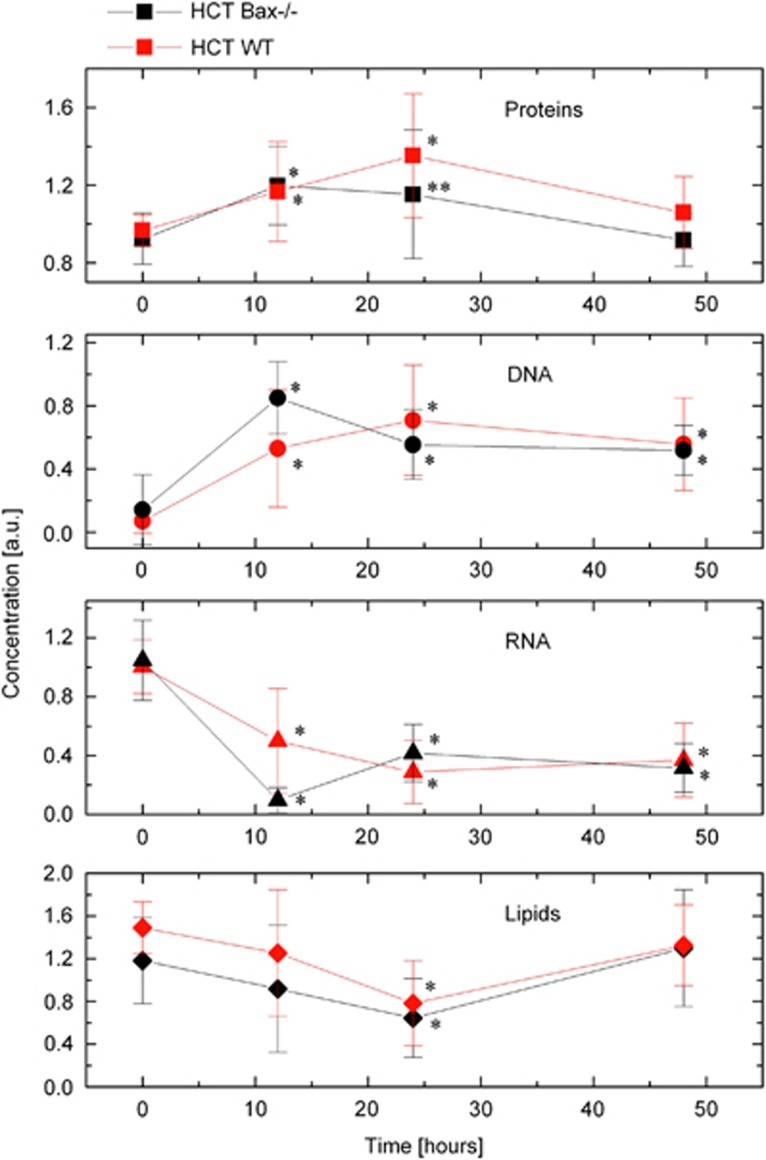
Dox induces accumulation of protein and DNA but not RNA and lipid on mitochondria. HCT116 WT and HCT116 Bax^−/−^ cells were unstimulated or treated with Dox (10 *μ*M) for indicated times. Relative levels of protein, DNA, RNA, and lipid on mitochondria during the apoptosis in response to Dox treatment are presented. Black lines represent the levels of macromolecules from HCT116 Bax^−/−^ cells. Red lines represent the levels of macromolecules from HCT116 WT cells. HCT, HCT116. Data are mean±S.D., *n*≥7; **P<0.05; *P<0.01

To interrogate the spectral differences observed during the course of Dox treatment, and to establish the contribution of protein, DNA, RNA, and lipid into the spectra of mitochondria, we applied a biomolecular component analysis–linear combination spectral modeling (BCA–LCSM) approach. By matching the measured Raman spectra and the model spectra (computer-generated linear combination of the concentration-calibrated reference spectra of protein, RNA, DNA, and lipid), the concentrations of these types of macromolecules in mitochondria were determined.^9,16^ In untreated cells, our findings demonstrated identical concentrations of protein, DNA, and RNA in molecular makeup of mitochondria in WT and Bax-deficient cells (Figure 2, top three panels). Surprisingly, lower level of lipids was observed in Bax-deficient as compared with WT-untreated cells (Figure 2, lowest panel).

### Dox induces accumulation of protein and DNA, whereas the levels of RNA and lipid were decreased at early time periods

To dissect the transformations of macromolecular structure of mitochondria upon DNA-damage-induced cell death, we quantified the levels of protein, DNA, RNA, and lipid during the course of Dox treatment. Remarkably, we found that molecular makeup associated with mitochondria is dynamically changed in response to Dox treatment. Moreover, WT and Bax-deficient cells showed a different dynamics of these transformations. Specifically, we observed accumulation of proteins early during the first 12 h after Dox treatment in both types of cells, but in WT cells, the levels of proteins continued to increase until 24 h after Dox treatment. Concentrations of protein were back to pre-treatment levels during late phase (i.e., ~48 h) of apoptosis in both types of cells (Figure 2). The levels of both RNA and lipids were decreased during first 24 h of treatment. The levels of RNA were maintained at decreased level, whereas lipid levels were back to pre-treatment levels at later stage during apoptosis in both cell types (Figure 2).

Dox induces DNA damage, and mitochondria also contain its own genome,^17,18^ suggesting that similar to nuclear genome, mtDNA may also be susceptible to damage and may affect the levels of mtDNA. Importantly, our findings demonstrated ~7-fold increase in the levels of DNA on mitochondria of HCT116 WT cells, which surprisingly remained elevated during apoptosis. Similarly, the levels of DNA on mitochondria were also higher in HCT116 Bax-deficient cells (Figure 2). These findings suggest that DNA-damaging agents promote mitochondria activation probably by upregulating the levels of mtDNA.

### Structural changes in mitochondrial proteins during DNA-damage-induced apoptosis

Mammalian mitochondria contain 1000–1500 different proteins that are collectively referred to as the ‘mitochondrial proteome'.^19^ Currently, there is limited knowledge on the dynamics and scale of the transformation of the mitochondrial proteome during apoptosis. Remarkably, Raman microspectroscopy provides a unique opportunity for quantitative analysis of secondary conformations prevailing in the mitochondrial proteome at any given time. To understand whether Dox treatment selectively modulates the mitochondrial protein conformations, we determined the intensities of vibrational frequencies produced by basic secondary structures such as *α*-helix, *β*-sheet, and random coil. For selective analysis of protein conformations, we adopted a modification of BCA–LCSM approach as described in Materials and Methods section. The normalized Raman spectral profiles assigned to the entire population of mitochondrial proteins were generated. To estimate patterns of secondary conformations of proteins populating at mitochondria, the spectra of bovine serum albumin (BSA) were subtracted from the spectra representing mitochondrial proteins (both normalized to phenylalanine Raman peak at 1004/cm).^20,21^ BSA is a well-characterized protein with ~55% *α*-helix and ~45% random coil conformations.^22^ BSA subtraction demonstrated negative peaks at the vibrations stretch of 930–950/cm and a band centered at 1652/cm, which are known to associate with *α*-helical secondary conformations of proteins. The presence of these negative bands at the difference spectra indicated significantly lower density of *α*-helix conformations in mitochondria proteome compared with BSA. The vibration bands centered at 1245 and 1682/cm are associated with random coil conformations, and the positive peak at the difference spectra indicated higher content of random coil protein conformation in the proteome of mitochondria compared with BSA. The lack of *β*-sheet motifs in BSA suggested that the differential spectra showing major positive bands at the stretch 1228–1240/cm and band centered at 1652/cm are assigned to *β*-sheet vibrational frequencies. We applied the same approach to analyze whether the concentration of all protein species is changed uniformly over the course of apoptosis, or if concentration changes can vary between individual proteins in mitochondria.

**Figure 3:**
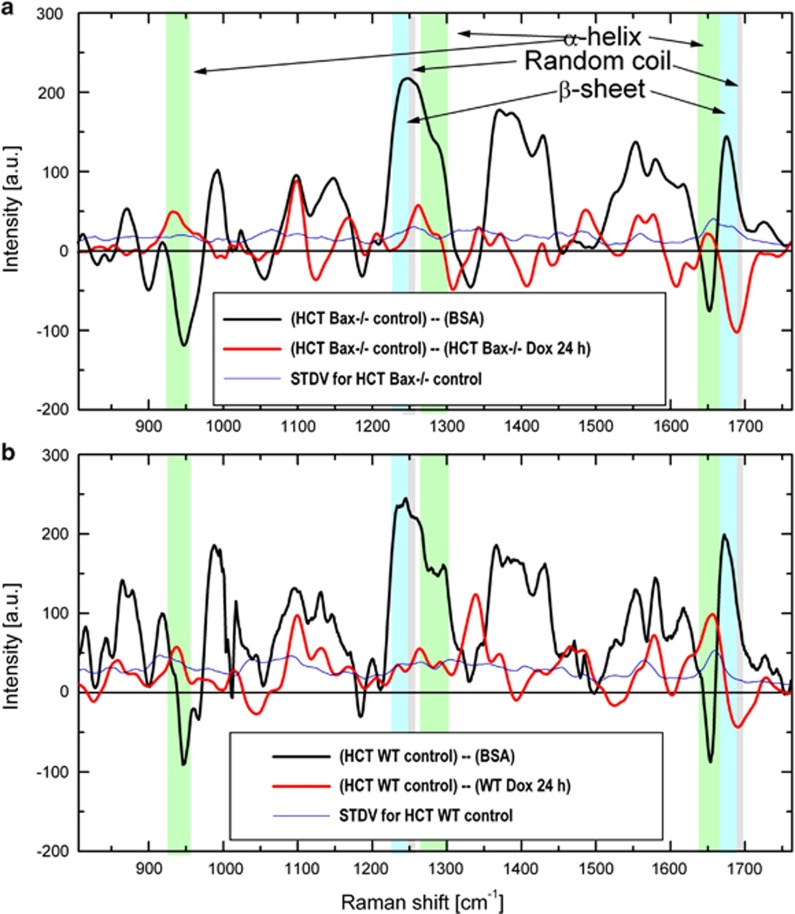
Dox decreases ratio of *α*-helix and increases *β*–sheets and random coil protein conformations on mitochondria. (**a** and **b**) HCT116 Bax^−/−^ and HCT116 WT cells were unstimulated or treated with Dox (24 h, 10 *μ*M). Black lines represent the spectral profiles obtained by subtraction of normalized Raman spectra of BSA from those of mitochondria proteins in control untreated HCT116 Bax^−/−^ (**a**) and HCT116 WT (**b**) cells. Red lines correspond to the spectral profiles obtained by subtraction of normalized Raman spectra of mitochondria proteins in Dox-treated HCT116 Bax^−/−^ (**a**) and HCT116 WT (**b**) cells from untreated control cells. S.D.'s for mitochondria proteins of HCT116 Bax^−/−^ and HCT116 WT cells are shown in blue color. Difference spectrum reveals significant changes for vibrational bands assigned to specific protein conformations (arrows pointing to the colored bands). Dox, doxorubicin; HCT, HCT116

**Figure 4:**
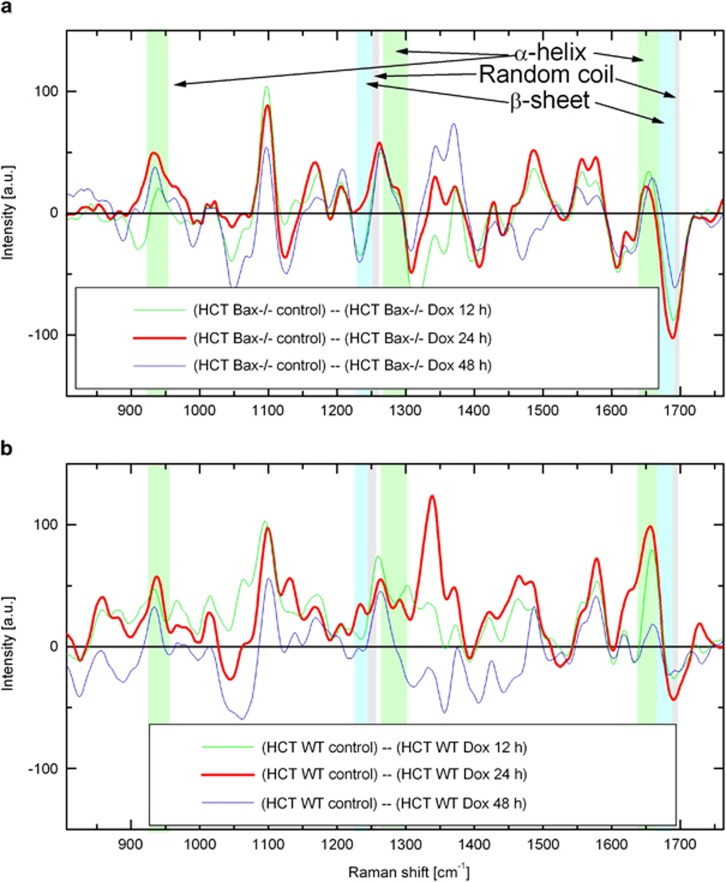
Dynamics of protein conformation changes during Dox treatment. (**a** and **b**) Difference of normalized Raman spectra of protein in mitochondria averaged over HCT116 Bax^−/−^ (**a**) and HCT116 WT (**b**), and randomly chosen cells as described in Materials and Methods section. Green, red, and blue lines represent differences in mitochondria proteins between untreated and treated at 12, 24, and 48 h, respectively, in HCT116 Bax^−/−^ (**a**) and HCT116 WT (**b**). Dox, doxorubicin; HCT, HCT116

To understand the changes in secondary structure, the spectra representing mitochondrial protein at 24 h of Dox treatment were subtracted from the protein spectra of non treated cells to generate a difference spectrum (Figure 3 and Supplementary Figure S2). In this case, an increase in any protein conformation content would be represented with a negative signal intensity of corresponding band in the difference spectrum while a decrease would be a positive signal. The differential spectra analysis demonstrated significant decrease of *α*-helix conformations following drug treatment for 24 h in both HCT116 WT and Bax-deficient cells (Figures 3a and b; see positive peaks at 930–950/cm and band centered at 1652/cm). At the same time, there is a strong kink (abrupt change from positive to negative) in the 1640–1700/cm vibration stretch signifying an increase in *β*-sheet and random coil Raman signals in the Dox-treated cells (Figures 3a and b). Similar trends were observed at 12 and 48 h after Dox treatment in both WT and Bax-deficient cells (Figures 4a and b).

### Activation of mitochondria is associated with higher levels of mtDNA in both WT and Bax-deficient cells upon Dox treatment

**Figure 5:**
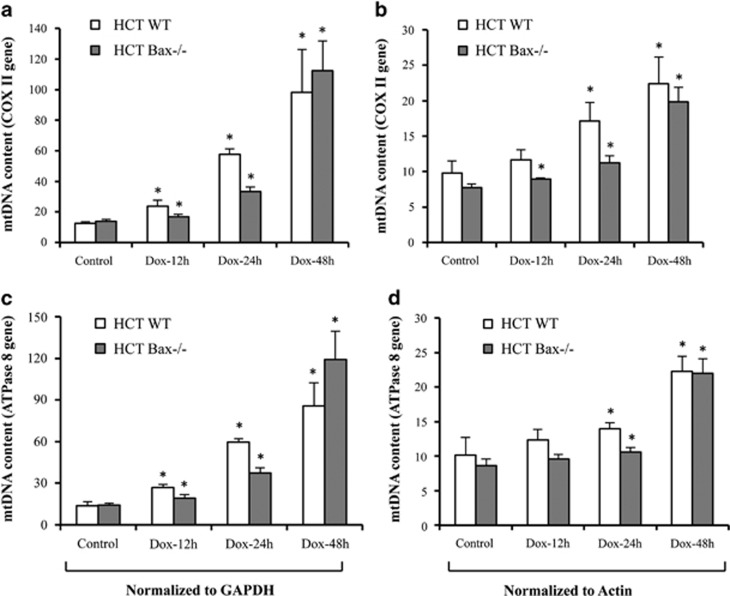
Dox enhances the levels of mtDNA. (**a**–**d**) ATPase 8 and cytochrome *c* oxidase subunit II (COX II) genes encoded by the mitochondrial genome was amplified and quantified by real-time PCR using the SYBR green chemistry on the Applied Biosystems 7300 real-time PCR system. Total DNA was extracted from HCT116 WT and HCT116 Bax^−/−^ cells treated with DMSO or Dox (10 *μ*M) for indicated times. COX II (**a** and **b**) or mitochondrial ATPase 8 (**c** and **d**) gene-specific primers were used to amplify, and values were normalized to actin or GAPDH. Data are mean±S.D., *n*=3; *P<0.01 as compared with DMSO-treated cells. Dox, doxorubicin; HCT, HCT116

To understand whether higher level of DNA accumulation on mitochondria during apoptosis is due to increased mtDNA, we investigated the level of mtDNA upon Dox treatment. The levels of mtDNA-encoded cytochrome *c* oxidase subunit II (COX II) and ATPase 8 (mitochondrially encoded ATP synthase subunit 8) gene were measured and normalized with nuclear genes actin and glyceraldehyde 3-phosphate dehydrogenas (GAPDH). We observed that the levels of mtDNA were significantly upregulated in WT cells starting at 12 h after Dox treatment. In general, Dox enhanced the levels of mtDNA in both types of cell with maximum increase observed at 48 h after treatment (Figures 5a–d).

### Increased levels of mtDNA correspond with increased cellular ROS and mitochondrial ROS production

**Figure 6:**
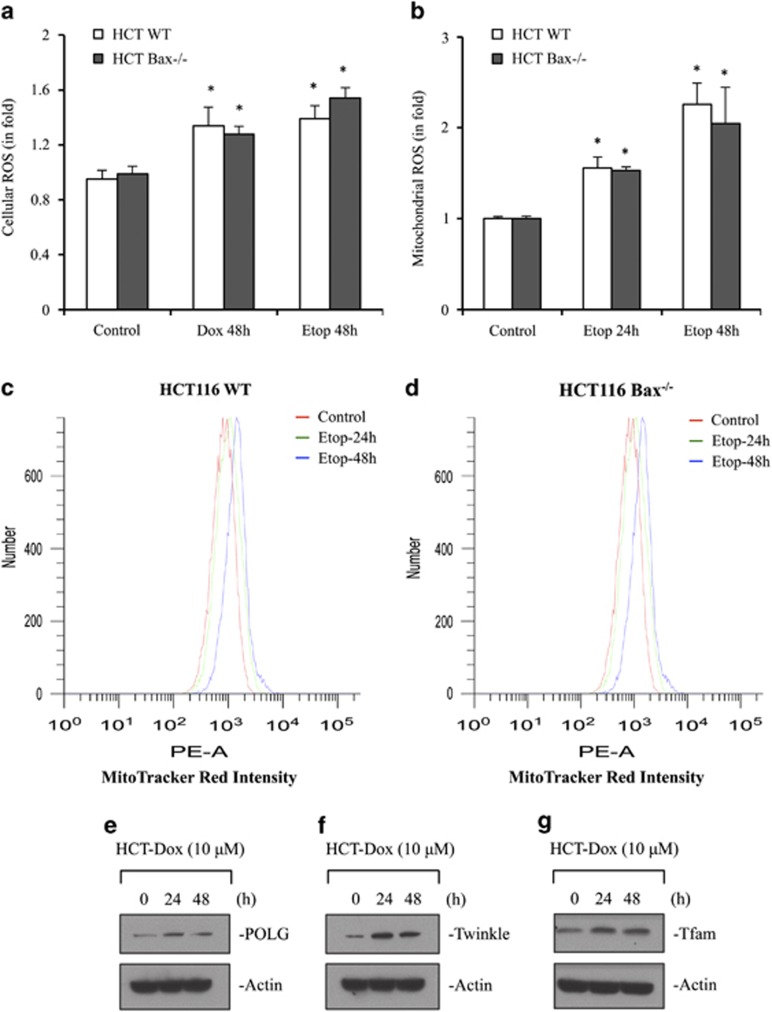
DNA-damaging agents induce cellular ROS, mitochondrial ROS, and expression of proteins required for mtDNA replication and its transcription. (**a**) HCT116 WT and HCT116 Bax^−/−^ cells were treated with DMSO or Dox (10 *μ*M) or etoposide (10 *μ*M) for 48 h. Cellular ROS were determined using flow cytometer after incubation with dihydrorhodamine 123 (DHR123). (**b**–**d**) HCT116 WT and HCT116 Bax^−/−^ cells were treated with DMSO or etoposide (10 *μ*M) for indicated times. Mitochondrial ROS were measured using flow cytometer after incubation with mitochondria-specific dye, MitoTracker Red (CM-H_2_XRos). (**e**–**g**) HCT116 WT cells were treated with Dox (10 *μ*M) for the indicated time intervals. After treatment, cells were harvested and equal amount of proteins were analyzed by western blot for quantification of protein level of POLG (**e**), Twinkle (**f**), Tfam, mitochondrial transcription factor A (**g**). Actin was used as a normalization control. Data (**a** and **b**) are mean±S.D., *n*=3; *P<0.05 as compared with DMSO-treated cells. Representative graphs of mitochondrial ROS are shown in **c** and **d**. HCT, HCT116; Etop, etoposide; Dox, doxorubicin; number, number of cells or number of events

As ROS signaling has an important role in mitochondrial biogenesis,^23^ we examined whether increased levels of mtDNA are associated with ROS accumulation at mitochondria. We first measured the levels of cellular ROS using dihydrorhodamine 123 (DHR123) upon treatment with DNA-damaging agents Dox and etoposide. We observed increased levels of cellular ROS at 48 h after Dox or etoposide treatment in both HCT116 WT and Bax-deficient cells (Figure 6a). Upregulation of mtDNA suggested that the activation of mitochondria could lead to production of higher mitochondrial ROS, we also determined the levels of mitochondrial ROS using MitoTracker CM-H_2_XRos and MitoSox Red. As Dox fluorescence spectrum overlaps with MitoTracker CM-H_2_XRos and MitoSox Red, we have used another DNA-damaging agent, etoposide. The mitochondrial ROS was significantly increased as compared with untreated cells in both WT and Bax-deficient cells starting at 3 h and onward in response to etoposide; however, only 24 h and 48 h data are shown (Figures 6b–d).

### Dox treatment induces genes responsible for mtDNA replication and transcription

**Figure 7:**
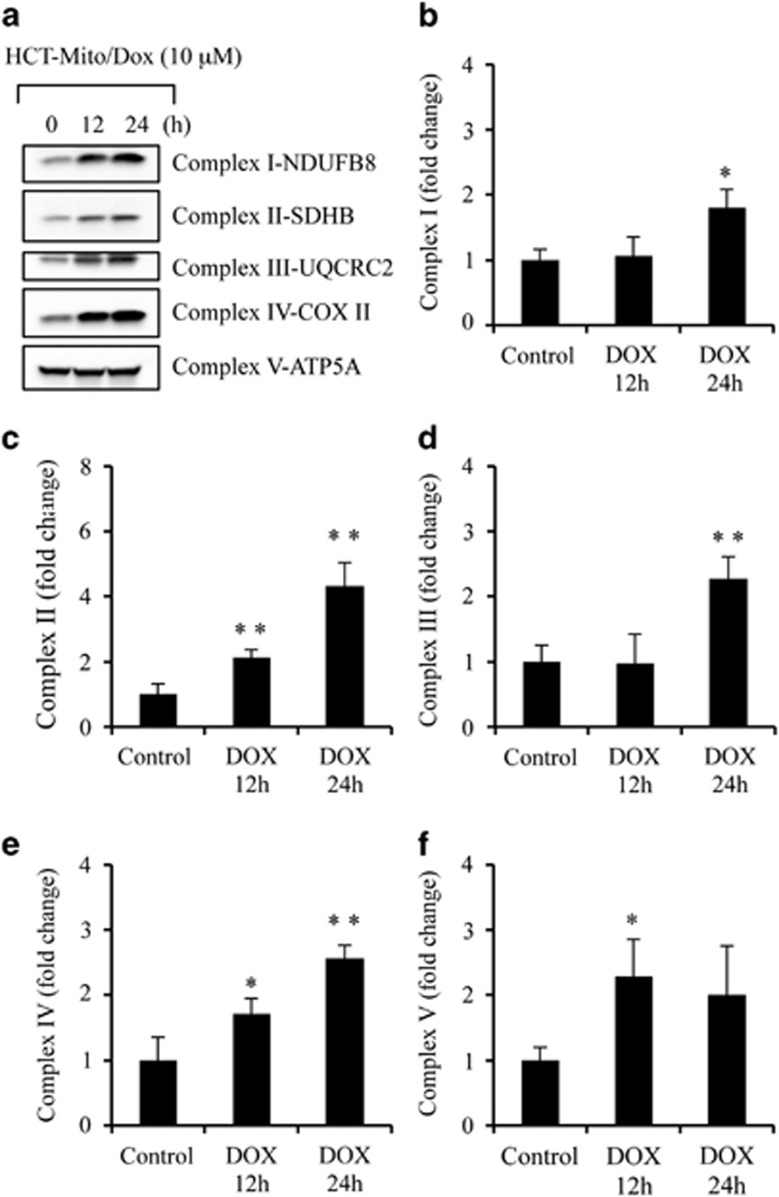
DNA-damaging agent Dox induces expression of OXPHOS proteins and enhances activities of OXPHOS complex. (**a**) Mitochondria isolated from untreated and Dox (10 *μ*M for 12 and 24 h)-treated HCT116 WT cells were subjected to western blotting for OXPHOS proteins. (**b**–**f**) Mitochondria isolated from HCT116 WT cells treated with Dox (10 *μ*M) for 0, 12, and 24 h were lysed by three freeze-thaw cycles in hypotonic buffer. Equal amount of mitochondria were assayed to determine enzymatic rate of respiratory complexes. Specificity of all enzymatic rates was controlled by using specific enzyme inhibitors or negative controls in parallel reactions. (**b**–**f**) The fold change of specific activity of OXPHOS complexes (nmol/min/mg mitochondrial proteins). Data are mean±S.D., n=3, *P<0.05 and **P<0.01 compared DMSO-treated cells. Complex V-ATP5A serves as loading control. Dox, doxorubicin; Mito, mitochondria; HCT, HCT116

Increased mtDNA levels and ROS production prompted us to ask whether the factors responsible for mtDNA replication or synthesis are also upregulated. To address this question, we determined the levels of polymerase-*γ* (POLG), the only polymerase responsible for mtDNA replication.^24^ We observed increased levels of POLG upon Dox treatment (Figure 6e). As POLG interacts with mtDNA helicase Twinkle to form functional replisome for proper functioning of mtDNA replication,^25^ we also investigated the level of Twinkle. Similar to POLG, the level of Twinkle was also upregulated (Figure 6f). Mitochondrial transcription factor A (Tfam) has an essential role in mtDNA transcription initiation.^26,27^ The increased level of Tfam upon Dox treatment (Figure 6g) demonstrates that machinery for transcription of mtDNA-encoded proteins is activated. Indeed, the level of mtDNA-encoded cytochrome *c* oxidase IV subunit II increased upon Dox treatment (Figure 7a), suggesting that newly synthesized mtDNA are functional in upregulating protein required for the proper functioning of the complexes of oxidative phosphorylation (OXPHOS).

### Dox induces expression of OXPHOS proteins and enhances the activity of OXPHOS complexes

Increased mtDNA synthesis and upregulation of mtDNA-encoded proteins suggest that Dox treatment activates OXPHOS function. To evaluate whether Dox modulates OXPHOS, we first measured the levels of OXPHOS proteins encoded by either nuclear DNA (nDNA) or mtDNA. We observed that Dox induced accumulation of proteins required for the organization of complex I–IV, whereas no changes were observed with protein related to complex V (Figure 7a). Further, we measured the activities of OXPHOS complexes (I, III, IV, and V) that require proteins encoded by mtDNA, as well as the activity of complex II, which is exclusively encoded by nDNA.^27^ We observed significant increase in OXPHOS complex activities compared with control HCT116 WT cells (Figures 7b–f). These finding suggest that DNA-damaging agents enhance mitochondrial respiration to execute apoptotic cell death.

## Discussion

Our findings for the first time provide a link between macromolecular structural landscape of mitochondria and progression of apoptosis. What is the significance of such macromolecular changes at the mitochondria during apoptosis? There are multiple studies dissecting cellular signaling focusing on either protein, lipid, RNA, or DNA. For example, how cytochrome *c* is release from mitochondria,^28, 29, 30, 31^ how lipid such as cardiolipin interacts with proapoptotic proteins to facilitate cytochrome *c* release.^32, 33, 34^ Similarly, RNA such as tRNA binds with cytochrome *c* and inhibits apoptosis.^35^ But these events require interactions between macromolecules, suggesting that there is an urgent need to dissect the involvement of various macromolecules such as protein, lipid, RNA, and DNA during apoptosis. Our findings define the changes in all major macromolecules during DNA-damage-induced apoptosis in cells. Although further studies are required to validate the importance of each macromolecule, this study provides the first step toward understanding the role of global changes in macromolecules during apoptosis.

Mitochondrion is the key-signaling center for cell survival and apoptosis; therefore, increased accumulation of proteins during apoptosis may represent a prosurvival mechanism. The elevated levels of protein could be due to increased synthesis of proteins encoded by mtDNA such as multiple subunits of cytochrome *c* oxidase, ATPase 8, and other subunits of proteins participating in the mitochondrial respiratory function to meet the increased need for ATP synthesis. Indeed, cDNA expression analysis of mitochondrial respiratory proteins shows many fold increase in mitochondrially localized or mtDNA-encoded proteins (data not shown). Multiple evidences suggest upregulation of protein essential for mitochondria functions.^36, 37, 38, 39^ In addition to mitochondrial genome-encoded proteins, multiple proteins encoded by nuclear genome also have role in energy-generating function of mitochondria. For example, cytochrome *c* also has been reported to be upregulated during apoptosis.^36,37^ Interestingly, not only protein having role in regulating mitochondria function, multiple other proapoptotic proteins are also translocated to mitochondria, thereby suggesting a dynamic balance between prosurvival and prodeath signaling in response to stress.^37,40, 41, 42^

What are global protein structure changes in mitochondria during stress such as in response to DNA damage? Accumulation or depletion of proteins in mitochondria suggests that during stress mitochondria may retain/sequester proteins of certain conformational structure either to restore mitochondria structure/function or to favor mitochondrial apoptosis. Our findings suggest that cancer cell mitochondria favors the accumulation of *β*-sheets/random coils over *α*-helix during DNA-damage-induced cell death. Although the underlying mechanisms of increased levels of *β*-sheets/random coils and reduction of *α*-helix require further comprehensive analysis, it is possible that DNA-damaging agents modulate mitochondrial folding machinery such as heat-shock protein 60 (Hsp60)-mediated protein folding. Hsp60 and its co-chaperone heat-shock protein 10 (Hsp10) have important role in mitochondrial protein folding.^43^ For example, Hsp60 favors interaction with hydrophobic amino acids, whereas in the presence of Hsp10, Hsp60 strongly associates with hydrophilic amino acids to facilitate protein folding.^44,45^

Primary function of upregulation and translocation of proapoptotic proteins such as Bim, t-Bid, Bax, and p53 to mitochondria from the cytosol is to induce mitochondria dysfunction leading to cytochrome *c* release and apoptosis.^7,46^ These proapoptotic proteins undergo conformational change that facilitates oligomerization and integration into the OMM. Although multiple mechanisms have been proposed to understand whether pore-forming proteins, Bax and Bak, are required to be in a particular conformation, the exact nature of these proteins are still not well defined.^28,29,31,47^ Multiple evidences support that *α*-helix conformation of multidomain Bax and Bak are critical for insertion to OMM and their oligomerization;^29, 30, 31^ however, whether *α*-helix conformation of proapoptotic proteins is maintained during apoptosis in live cells is not clearly defined. Because lipid levels and composition undergo dynamic change during apoptosis,^32, 33, 34^ the conformation of OMM-integrated or OMM-attached proteins, including Bax and Bak, are likely to be affected by lipid dynamics on mitochondria. Whether our findings on reduced levels of *α*-helix along with increased *β*-sheets and random coil are directly applicable to Bax and Bak conformation changes need further investigation; however, significantly reduced levels of lipid on mitochondria are likely to influence the conformation of proapoptotic proteins.

What are the secondary messengers or signaling molecules that may participate in modulating the global structural changes on mitochondria? ROS function as key-signaling molecules in the proliferation and elevation of mitochondrial biomass.^23,48^ Indeed, our findings demonstrated that DNA-damaging agents including Dox and etoposide induce total cellular and mitochondrial ROS production. The increased ROS may contribute to the mitochondrial nuclear cross talk causing synthesis of mtDNA leading to the increase in mitochondrial biomass, which may lead to the activation of mitochondrial respiration. Our findings support this notion that DNA-damaging agents induce ROS that activate expression of proteins required for mtDNA replication and transcription of mtDNA-encoded protein. Since functional mitochondrial respiration is critical for apoptosis induction, increased mitochondrial respiratory function may be one of the causes of apoptosis induction in response to Dox. Indeed, increased levels of mitochondrial biomass (data not shown) accompanied by increased activity of OXPHOS complexes indicate that DNA-damaging agents including Dox and etoposide activate mitochondrial function causing restoration of apoptosis. The process of apoptosis is often associated with mitochondrial fission, and mitochondrial fission is linked to the maintenance of mtDNA.^49,50^ Therefore, during DNA-damage-induced apoptosis, increased mitochondrial fission may ultimately lead to the activation of mtDNA synthesis machinery causing increased mtDNA copy number. Indeed, exposure of cells to DNA-damaging agent Dox induces the expression of POLG and Twinkle.

The increased mitochondrial ROS may also participate in peroxidation of lipids such as cardiolipin, subsequently leading to disruption of lipid structure and cristae remodeling.^51,52^ As cristae accommodate significant amount of lipid structure in mitochondria, their disruption may contribute to the reduction of lipid levels during apoptosis.^52^ ROS could also target RNAs leading to the degradation or reduction of RNA on mitochondria.^53^ Increased levels of proteins on mitochondria upon Dox treatment also infer that RNA synthesis may not have proportionally increased, causing exhaustion of RNA pool in mitochondria.

Together, we have demonstrated that Raman microspectroscopy can distinguish dynamic distribution of macromolecules including proteins, DNA, RNA, and lipids in unstimulated resting and apoptotic cells in response to DNA-damaging agents. These type of measurements will allow us to understand the dynamic response of macromolecules without any invasive methods during apoptosis. In addition, this will also help determine secondary structure of proteins, which may have significance in understanding the physiological states of cancer stem cells, cardiomyocytes, and neurons during stress, even if only few cells are available for the study. Raman microspectroscopy allows quantitative probing of dynamic macromolecular landscape at the single organelle level, and thus identifying the mitochondrial function at normal physiological and stress conditions. As the process of apoptosis is highly complex and proceeds with variable dynamics, even in genetically identical cells, the identification of universal structural hallmarks expressed at particular stages of apoptosis would be highly advantageous to study this process in real time. Vibration spectroscopy fingerprinting of proteins, DNA, RNA, and lipids macromolecules in mitochondria as reported here may identify a potential marker for determining the physiological state of cells. As Raman spectroscopy characterization is applicable for small cellular populations as well as for single-cell models, further advancement of this technique could lead to high-throughput screening for modulators of mitochondrial dysfunction associated with multiple diseases such as cancer, cardiac, and neurodegenerative disorders.

## Materials and Methods

### Cells and reagents

Isogenic HCT116 WT and HCT116 Bax^−/−^ colon cancer cells were kindly provided by Dr. B Vogelstein^54,55^ and cultured in McCoy's 5A medium supplemented with 10% FBS. The primary antibodies against cytochrome *c* (monoclonal antibody, mAb) were purchased from BD Biosciences (San Jose, CA, USA). COX II (Abcam, Cambridge, MA, USA), Hsp60 (EMD Millipore, Cambridge, MA, USA), and actin (mAb; BD Biosciences) were obtained from the indicated suppliers. Anti-Tfam, rabbit monoclonal anti-DNA POLG, and anti-Twinkle antibodies were purchased from Abcam. MitoProfile Total OXPHOS human WB Antibody cocktail (Abcam) was used as primary antibodies for western blot analysis.

Secondary antibodies and ECL reagents were acquired from GE Healthcare (Pittsburgh, PA, USA). MitoTracker Green, MitoTracker Orange, CM-H_2_XRos, MitoSox, and DHR123 were purchased from Thermo Fisher Scientific (Carlsbad, CA, USA). The fluorogenic caspase-3 substrate DEVD-AFC was obtained from Enzo Life Sciences (Farmingdale, NY, USA). All other chemicals were purchased from Sigma Chemical Company (St. Louis, MO, USA) unless specified otherwise.

### Whole-cell lysate preparation, subcellular fractionation, and western blotting

Preparation of whole-cell lysates, mitochondrial and cytosolic fractions, and western blotting were performed as mentioned previously.^37,56^

### Quantification of apoptosis and caspase activities measurement

Harvested cells were labeled with Trypan blue dye to quantify both live and dead cells. DEVDase activities were measured as described previously.^37,56^

### Immunofluorescence

Cells grown on coverslips were treated with Dox, and 15 min before the end of treatment cells were incubated live with DAPI and MitoTracker Orange to label both nuclei and mitochondria, respectively.^37,40^

### Mitochondrial respiratory complex enzyme activity measurement

Mitochondria were isolated from Dox-treated or -untreated cells. Equal amounts of mitochondria were subjected to three freeze-thaw cycles, and 25–50 *μ*g/ml mitochondrial proteins were used for OXPHOS complex activities as described previously.^57,58^ All reactions were carried out at 30 °C in a reaction volume 100 *μ*l using a Beckman spectrophotometer (Brea, CA, USA).

Complex I (NADH ubiquitin oxidoreductase) activity was determined by measuring the rotenone-sensitive NADH oxidation at 340 nm using coenzyme Q1 as an electron acceptor. Complex II (succinate dehydrogenase) activity was determined by measuring reduction rate of 2,6-dichloroindolphenol in the presence and absence of coenzyme Q1. Complex III (decylubiquinol cytochrome *c* oxidoreductase) activity was determined by monitoring the antimycin A-sensitive reduction rate of cytochrome *c* in the presence of fully reduced CoQ_2_ as the electron source. Complex IV (cytochrome *c* oxidase) activity was measured by KCN-sensitive oxidation of cytochrome *c*. Complex V (ATP synthase) activity was determined by measuring oligomycin-sensitive oxidation rate of NADH at 340 nm.

### Analysis of mtDNA content by real-time PCR

The mtDNA levels were quantified as described previously.^59^ In brief, total DNA, containing both mtDNA and nDNA, was isolated from HCT116 WT and HCT116 Bax^−/−^ using the ZR Genomic DNA II Kit (Zymo Research, Irvine, CA, USA). After quantification of DNA samples by the NanoDrop8000 (Thermo Fisher Scientific), mtDNA content was determined on the Applied Biosystems 7300 real-time PCR system. *GAPDH* and *β-actin* or *ATPase 8* and *COX II* were used for amplification of nuclear or mtDNA, respectively. Primers for *GAPDH*, *β-actin, ATPase 8, and COX II* were used as described previously.^59,60^ *GAPDH* (forward): 5′-CCCCACACACATGCACTTACC-3′, *GAPDH* (reverse): 5′-CCTAGTCCCAGGGCTTTGATT-3′ *β-actin* (forward): 5′-TCACCCACACTGTGCCCATCTACGA-3′, *β-actin* (reverse): 5′-CAGCGGAACCGCTCATTGCCAATGG-3′ ATPase 8 (forward): 5′- AATATTAAACACAAACTACCACCTACC-3′, ATPase 8 (reverse): 5′-TGGTTCTCAGGGTTTGTTATA-3′ *COX II* (forward): 5′-CCCCACATTAGGCTTAAAAACAGAT-3′, *COX II* (reverse): 5′-TATACCCCCGGTCGTGTAGCGGT-3′. The real-time PCR was carried out in a total reaction volume of 10 *μ*l containing 5 *μ*l of 2 × iTaq SYBR Green Supermix with ROX (Bio-Rad, Hercules, CA, USA), 10 ng of template DNA, 500 nM each of forward and reverse primers, and nuclease-free water. A melting-curve analysis done at the end of amplification showed the absence of nonspecific amplification or primer dimer formation. The threshold cycle number (*C*_t_) values for each reaction were calculated using the 7300 system SDS software (Thermo Fisher Scientific). Standard curves generated from DNA obtained from untreated LNCaP cells using 10 ng to 10 pg provided PCR efficiency based on the equation *E*=10^(−1/slope)^−1.^61^ Average *C*_t_ values were obtained by amplification of *COX II* (mtDNA specific) and *β-actin (*nDNA specific). mtDNA content was determined as 2^Δ*C*t^ or fold difference of mtDNA from nDNA.^59,62,63^

### Measurements of cellular ROS and mitochondrial ROS using flow cytometry

To quantify cellular ROS, unstimulated or treated cells were washed and incubated with DHR123^64,65^ at 5 *μ*M in culture medium without serum at 37 °C for 30 min. For mitochondrial ROS measurement, we used ROS-specific probe, reduced MitoTracker Red (CM-H_2_XRos), and MitoSox red as described previously.^65,66^ Cells were suspended in growth medium without serum and were incubated with freshly prepared CM-H_2_XRos (0.5 *μ*M) or MitoSox red (500 nM) for 30 min at 37 °C.

### Raman microspectroscopy measurements

HCT116 WT and HCT116 Bax^−/−^ cells were grown in Mattek glass bottom dishes and were treated with Dox (10 *μ*M) upto 48 h at 37 °C. Fluorescence staining of mitochondria was performed using MitoTracker Green FM (Thermo Fisher Scientific) at a 100 nM concentration for 30 min. At the next step, the medium was changed, mitochondria were visualized, and Raman spectra of these organelles were acquired as described below.

### Raman microspectrometry: instrumentation, calibration, and data analysis

Our custom-made confocal Raman microspectrometer is based on an inverted Nikon TE200 microscope (Nikon, Melville, NY, USA) equipped with a He-Ne (Coherent, 632.8 nm, Santa Clara, CA, USA) excitation laser, fiber-input MS3501i imaging monochromator/spectrograph (Solar TII, Minsk, Belarus), and Hamamatsu S9974 series (Hamamatsu, Bridgewater, NJ, USA) CCD cooled down to −60 °C.^9,16^ This configuration provides the Raman spectral measurement within the spectral range of 600–3000/cm. The spectral resolution for the fixed diffraction grating position (wave number interval of 1210/cm) was ~1.5/cm. An excitation laser beam of ~30 mW power is focused onto the sample in a spot of~0.8 *μ*m using a × 100 NA=1.3 Nikon oil-immersion objective lens. To enable signal acquisition in a confocal mode, a 100*-μ*m pinhole was used. The confocal parameter was estimated to be ~1.8 *μ*m by measurement of z-position dependence of Raman signal in thin (~200 nm) polystyrene film spin-coated on glass substrate. To ensure the absence of vibration, thermal drift, or other motion in our system during experiments, we visually verified the XYZ position of the cell before and after each measurement. Our spectroscopic studies performed in live cells did not produce any visible changes in cellular morphology^9^ and showed no cytotoxicity by standard cell-viability tests.

All Raman spectra were preprocessed using background subtraction, Savitzky–Golay smoothing (second order of polynom and 13 points of smoothing), and baseline correction. Background elimination was performed by subtraction of the Raman spectra of incubation medium and background equalization of measured spectrum and corresponding model^16^ (Supplementary Figure S3). Raman spectral concentration calibration was performed using BSA, calf thymus DNA, *Saccharomyces cerevisiae* RNA, and bovine heart lipids; the details are provided in our earlier study.^9^ The number of measurements ranged from 7 to 21 cells for each studied experimental group of cells. The integration time for Raman spectral measurements was 180 s for all experiments. Each measurement was repeated three times, and the signal was averaged for noise reduction. The measured spectra showing unusually high concentrations of lipids (>50 mg/ml) were omitted from the analysis due potential input of lipid droplets in the mitochondria proximity. Statistical significance of difference for macromolecular concentrations in cells from different experimental groups was performed by one-way ANOVA test with *P*<0.05.

### BCA of mitochondria

Raman spectrometry analysis utilized BCA–LCSM.^9,10,67, 68, 69^ Raman spectra were acquired using confocal Raman spectroscopy setup in the central parts of mitochondria of cultured cells and the acquired spectra were processed as described above.

The model spectrum was simulated as a best fit to the experimental spectrum through a linear combination of weighted reference spectra of the basic classes of biomolecules such as protein, DNA, RNA, and lipid.^9^ A numerical value of the fractional contribution (weight) of each biomolecular component of the model Raman spectrum was assigned to the concentration of macromolecules in the mitochondria for further analysis. In our modeling approach, we have used the reference spectra of cellular DNA, RNA and lipid, the Raman spectra of calf thymus DNA, *S. cerevisiae* RNA, and bovine heart lipid extract. These spectra are regarded to be very close across the entire family of each type of biomolecules under diverse biological species.^9,16^ At the same time, the high diversity of mitochondrial proteins,^70^ with different composition and conformations, makes the selection of a universal standard for proteins a highly problematic task. In order to analyze the contribution of protein constituents to the measured Raman spectra, we adopted an approach described in our earlier study.^16^

Considering that (i) the Raman spectra of nucleic acids (DNA and RNA) and cellular lipids are relatively unchanged, and (ii) concentrations of RNA, DNA, and lipids in mitochondria are lower than that of proteins, a subtraction of the linear combination of the DNA, RNA, and lipids weighted reference spectra from the acquired Raman spectrum will produce a spectrum representing mostly proteins, and therefore, was approximated as the ‘mitochondrial proteins' spectrum. A major advantage of this approach for spectra analysis is that normalized Raman spectrum cleared from contributions of RNA, DNA, and lipid molecules reflects variations of remaining molecular constituents more clearly. Averaged by the number of measurements, this spectral profile may serve as an optimal model component of mitochondrial proteins. Besides proteins, this spectrum includes contributions of small organic and nonorganic molecules, as well as unaccounted variations of RNA, DNA, and lipid spectra. In our approach, we analyzed specificities of ‘mitochondrial proteins' spectra (instead of ‘residuals' for traditional LCSM method), utilizing the data for most known vibration frequencies assignments of biomolecules. In this analysis, spectra were normalized to phenylalanine Raman peak at 1004/cm.

### Statistical analysis

Results are presented as mean±S.D. of data from at least three independent experiments. Statistical analysis was performed by ANOVA using Sigma Stat. Significant changes (*P*<0.05) are represented in figures by (*) symbol.

## Abbreviations

POLG: polymerase-*γ*
Tfam: mitochondrial transcription factor A
OXPHOS: oxidative phosphorylation
ROS: reactive oxygen species
BCA–LCSM: biomolecular component analysis (BCA) and a linear combination spectral modeling (LCSM)
DHR123: dihydrorhodamine 123
mtDNA: mitochondrial DNA
nDNA: nuclear DNA
BSA: bovine serum albumin
GAPDH: glyceraldehyde 3-phosphate dehydrogenase
Bcl-2: B-cell lymphoma 2
Bak: Bcl-2 antagonist/killer
Bax: Bcl-2-associated X protein
ATPase 8: mitochondrially encoded ATP synthase subunit 8
Hsp60: heat-shock protein 60
Hsp10: heat-shock protein 10
DOX: doxorubicin
OMM: outer mitochondrial membrane
