# Supplementary material for: Transformations of the macromolecular landscape at mitochondria during DNA-damage-induced apoptotic cell death
Source: Cell Death Dis. 2014 Oct 9;5(10):e1453–. doi: 10.1038/cddis.2014.405 (PMC4649512; doi:10.1038/cddis.2014.405)
Supplement: Supplementary Information [file cddis2014405x1.pdf]

Supplemental Figure Legends

<u>Figure S1</u>**: Bax-deficiency does not modulate the intensity of Raman spectra.** HCT116 WT and HCT116 $Bax^{-/-}$ cells were unstimulated or treated with doxorubicin (10 μM) for indicated times. Raman spectra of mitochondria sites averaged over HCT116 $Bax^{-/-}$ and HCT116 WT randomly chosen cells (n=7-20) with doxorubicin treatments. HCT, HCT116.

<u>Figure S2.</u> **Normalized Raman spectra of protein on mitochondria upon doxorubicin treatment.** HCT116 $Bax^{-/-}$ and HCT116 WT cells were unstimulated or treated with doxorubicin (10 μM) for indicated times. Normalized Raman spectra of protein on mitochondria averaged over HCT116 $Bax^{-/-}$ and HCT116 WT randomly chosen cells as described in Materials and Methods.

<u>Figure S3.</u> **Analysis of Raman spectra obtained in mitochondria of HCT116 WT control cells**. All Raman spectra were preprocessed for background subtraction, Savitzky-Golay smoothing (2nd order of polynom and 13 points of smoothing) and baseline correction. Background elimination was performed by subtraction of the Raman spectra of incubation medium and background equalization of measured spectrum and corresponding model. **(A)** Measured raw spectra; **(B)** preprocessed spectra; **(C)** mean spectrum for HCT116 WT control cells, corresponding model for mean curve and difference spectrum between spectrum and model; and **(D)** difference spectra between measured spectra and corresponding model for each individual cell in HCT116 WT control cells in culture.

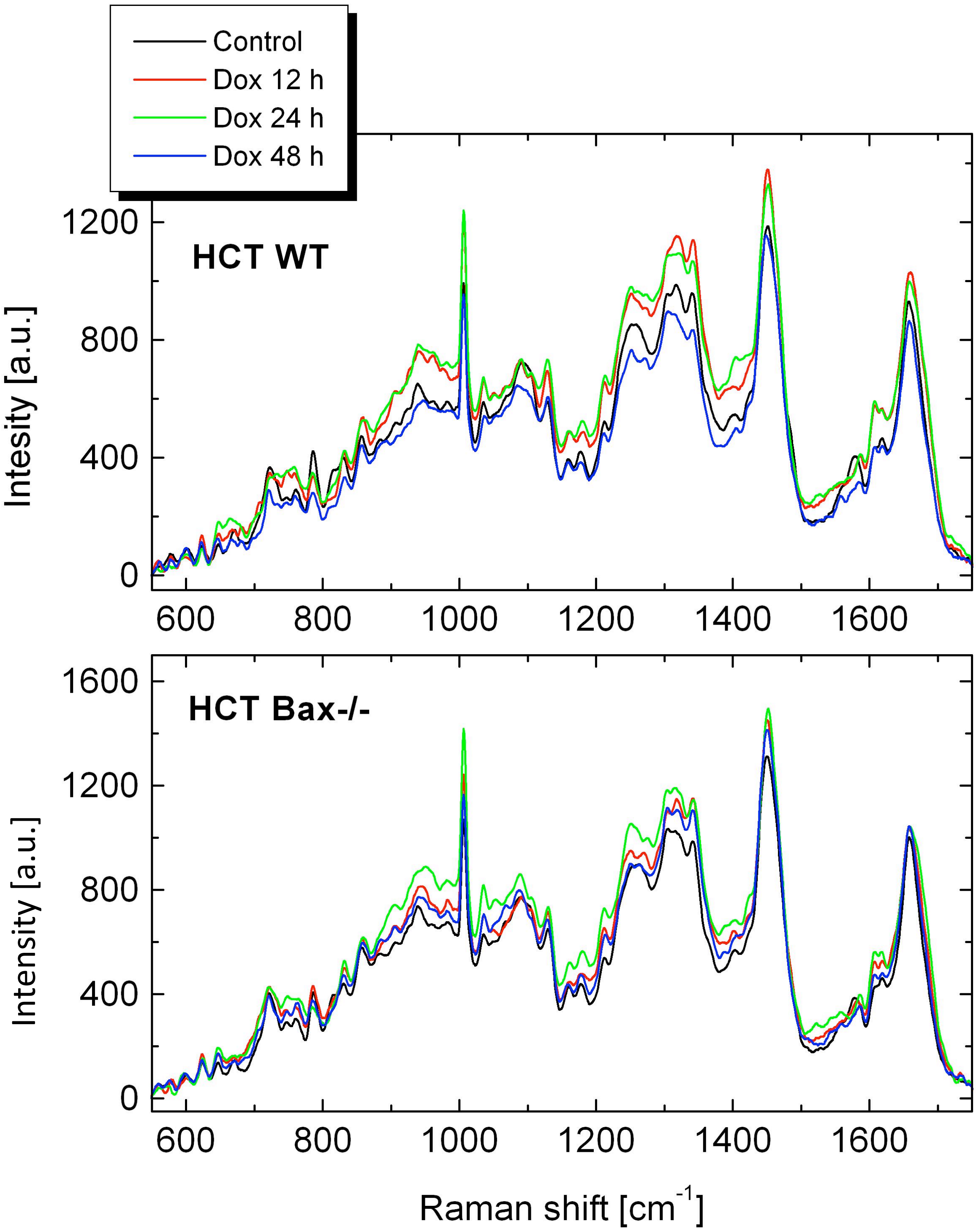
Control
Dox 12 h
Dox 24 h
Dox 48 h
HCT WT
Intesity [a.u.]
0
400
800
1200
600
800
1000
1200
1400
1600
HCT Bax-/-
Intensity [a.u.]
0
400
800
1200
1600
600
800
1000
1200
1400
1600
Raman shift [cm-1]

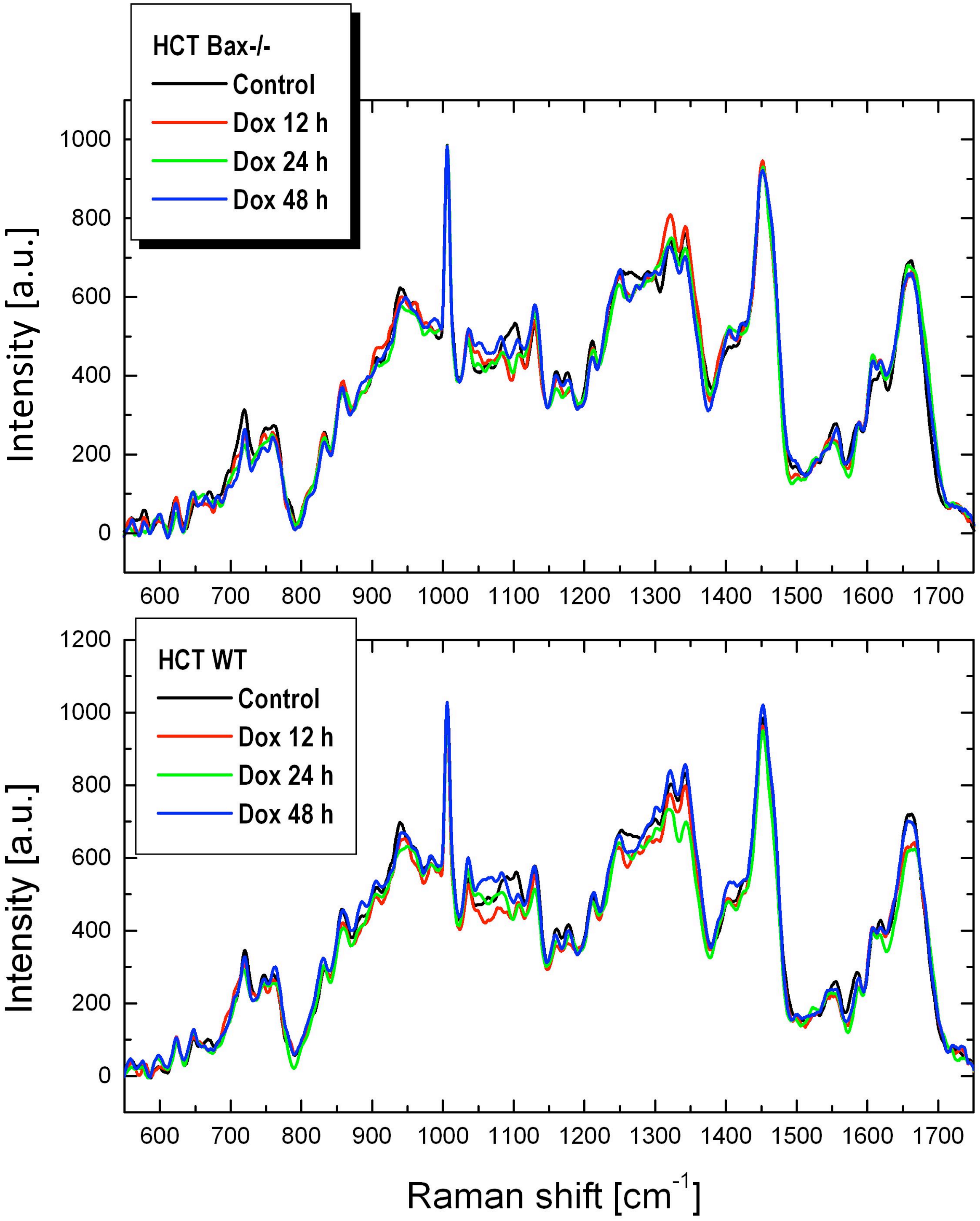
HCT Bax-/-
Control
Dox 12 h
Dox 24 h
Dox 48 h
Intensity [a.u.]
0
200
400
600
800
1000
600
700
800
900
1000
1100
1200
1300
1400
1500
1600
1700
HCT WT
Control
Dox 12 h
Dox 24 h
Dox 48 h
Intensity [a.u.]
0
200
400
600
800
1000
1200
Raman shift [cm-1]

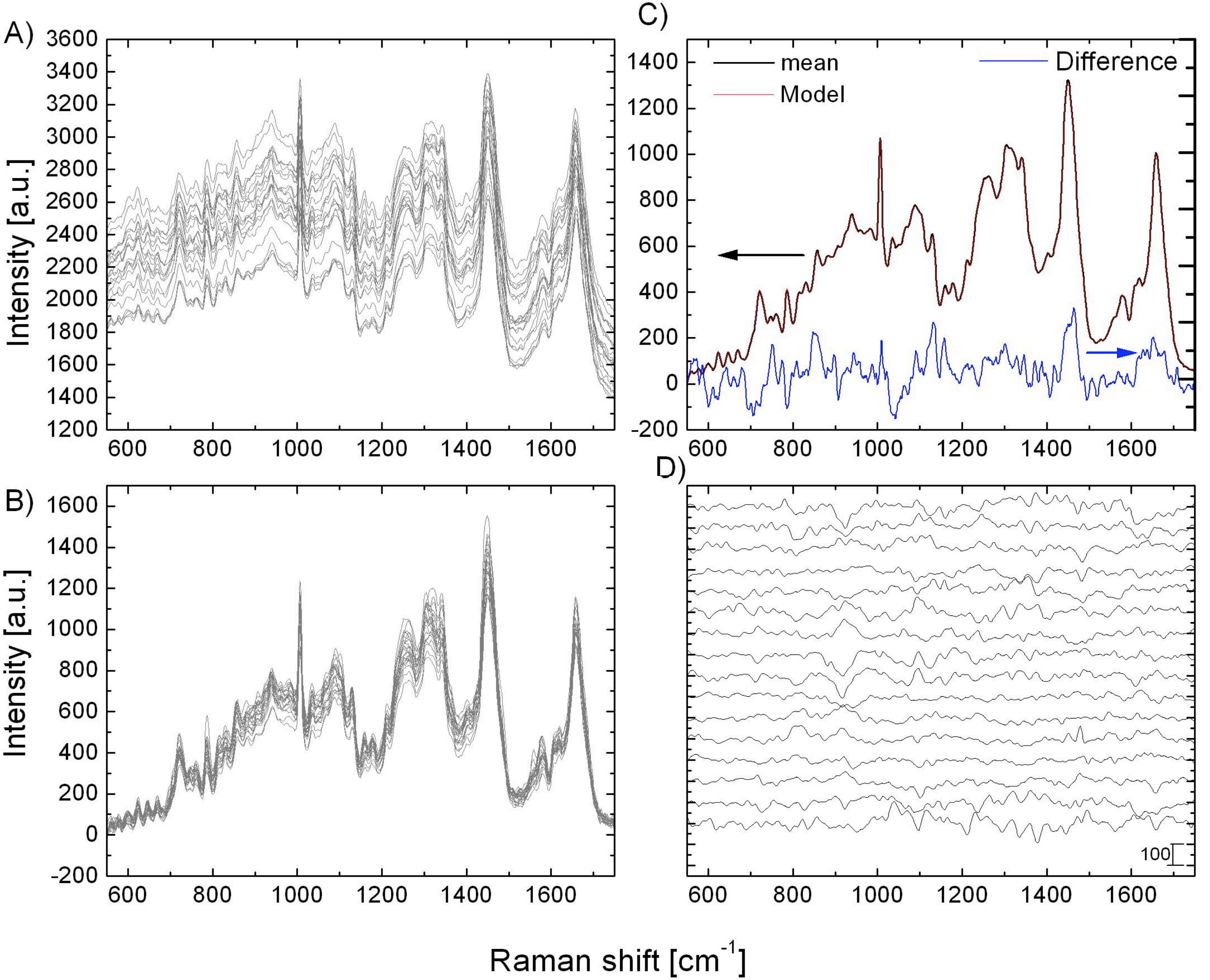
A)
B)
C)
D)
Intensity [a.u.]
Intensity [a.u.]
mean
Model
Difference
100
Raman shift [cm^{-1}]
